# Rare incidence of non-secretory myeloma with talaromycosis: a case report

**DOI:** 10.1186/s12879-021-06641-3

**Published:** 2021-09-16

**Authors:** Haiting Qin, Ye Qiu, Yanmei Huang, Mianluan Pan, Dong Lan, Wen Zeng, Jianquan Zhang

**Affiliations:** 1 Department of Respiratory Medicine, The Eighth Affiliated Hospital, Sun Yat Sen University, Shenzhen, 518000 Guangdong China; 2 Department of Respiratory and Critical Care Medicine, The First Affiliated Hospital of Guangxi Medical University, Nanning, 530021 Guangxi China

**Keywords:** *Talaromyces marneffei*, Non-secretory myeloma, Talaromycosis, Human immunodeficiency virus, Case report

## Abstract

**Background:**

*Talaromyces marneffei* (TM) primarily infects patients with co-morbidities that cause immunodeficiency, but non-secretory myeloma (NSMM) is rare. TSM and NSMM are associated with fever, osteolysis, and swollen lymph nodes, thereby making it difficult for clinicians to make differential diagnosis. In this case, we describe TM infection coexisting with NSMM.

**Case presentation:**

We retrospectively reviewed the case of a male (without human immunodeficiency virus infection) with fever, thoracalgia, swollen lymph nodes, and subcutaneous nodules who presented to the First Affiliated Hospital of Guangxi Medical University in February 2014. Chest computed tomography revealed patchy infiltration and positron emission tomography/computed tomography showed increased metabolic activity in the lower-right lung, lymph nodes, left ninth rib, and right ilium. Pathological examination of the lung, lymph nodes, subcutaneous nodules, and bone marrow showed no malignancy, he was diagnosed with community-acquired pneumonia. His clinical symptoms did not improve after anti-bacterial, anti-*Mycobacterium tuberculosis*, and anti-non-*M. tuberculosis* treatment. Later, etiological culture and pathological examination of the subcutaneous nodule proved TM infection, and the patient was re-diagnosed with disseminated TSM, which involved the lungs, lymph nodes, skin, bone, and subcutaneous tissue. After antifungal treatment, the patient showed significant improvement, except for the pain in his bones. Imaging showed aggravated osteolysis, and bone marrow biopsy and immunohistochemistry indicated NSMM. Thus, we conclusively diagnosed the case as NSMM with TSM (involving the lungs, lymph nodes, skin, and subcutaneous tissue). His condition improved after chemotherapy, and he was symptom-free for 7 years.

**Conclusion:**

TM infection is rare in individual with NSMM. Since they have clinical manifestation in common, easily causing misdiagnosis and missed diagnosis, multiple pathological examinations and tissue cultures are essential to provide a differential diagnosis.

## Background

Talaromycosis (TSM) is an invasive systemic fungal infection caused by *Talaromyces marneffei* (TM) around the skin, lymph nodes, lung, liver, bone, and bone marrow, which generally manifests as fever, cough, dyspnea, weight loss, skin and soft tissue lesions, hepatosplenomegaly, lymphadenopathy, and osteolysis. TM primarily infects patients with human immunodeficiency virus (HIV). However, in recent years, we have seen an increase in the number of TM-infected HIV-negative patients [1]. HIV-negative patients infected with TM are associated with co-morbidities, such as hematological malignancies, connective tissue disease, and diabetes mellitus [1, 2]. Non-secretory myeloma (NSMM) is a rare subtype (2–4%) within the multiple myeloma (MM) group of hematological malignancies. MM and NSMM share clinical and radiological features, both are characterized by osteolysis, anemia, hypercalcemia, and renal insufficiency; fever and swollen lymph nodes are also common. However, patients with NSMM exhibit normal serum and urine electrophoretic profiles [3]. Individuals with myeloma often present with opportunistic infections, but TM infection is rare. Both MM and TSM can manifest as fever, swollen lymph nodes, and osteolysis, which leads to misdiagnosis or missed diagnosis, resulting in a delay in treatment. In this report, we have described the clinical progression and treatment of a rare case of NSMM with TM infection in an HIV-negative patient.

## Case presentation

**Fig. 1:**
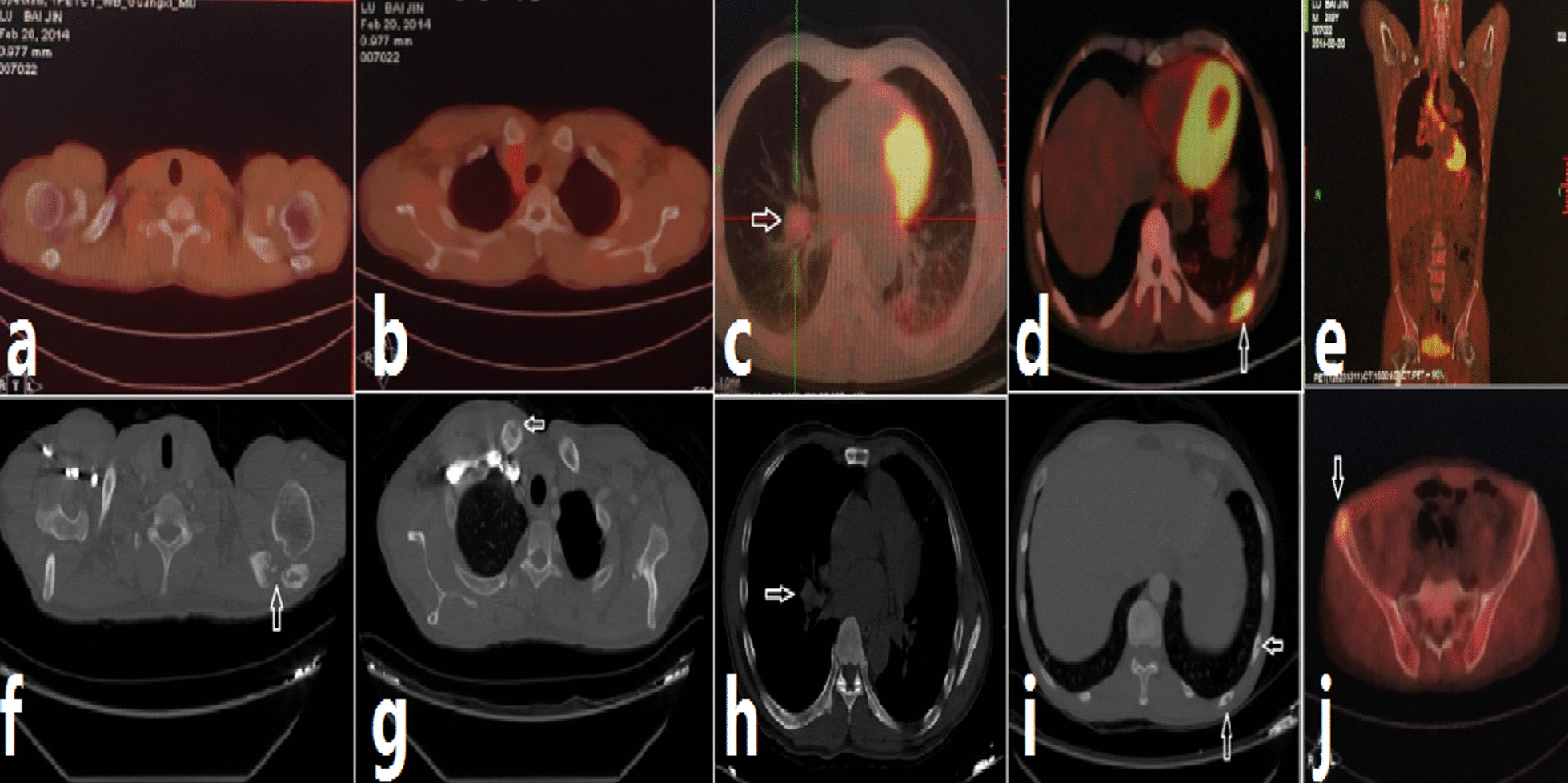
The comparison of imaging before and after receiving antifungal treatment. Positron emission tomography/computed tomography results showing the increase in metabolic activity in the right lower lung (**c**), mediastinal and hilar lymph nodes, ninth rib on the left (**d**), and right ilium (**j**). Standard uptake of 18F-fludeoxyglucose was 2.7, 8.0 (maximal), 6.5, and 4.2, respectively. After antifungal treatment (no chemotherapy), chest computed tomography showed well-defined aggravated osteolysis in the left acromion (**f**), clavicle (**g**), and ninth and tenth ribs on the left (**i**). Bone marrow cavities were also detected Panels (**a**), (**b**), (**h**) inside the image of Figure [1] are to compare the degree of osteolysis and lung infection in the same part of the patient before and after receiving antifungal treatment, so there is no redundant explanation. Panels (**e**) is a coronal view of PET/CT

A 51-year-old man presented with a 2-year history of intermittent fever, thoracalgia, swollen lymph nodes, and subcutaneous nodules and was admitted to the First Affiliated Hospital of Guangxi Medical University in February 2014. His medical, family, and psychosocial history including relevant genetic information had no special. On examination, he exhibited a maximum body temperature of 38.2 °C and multiple swollen lymph nodes, painful nodules were found in the right subclavicular region and left chest wall. Routine blood examination revealed an elevated leukocyte count of 25.43 × 10^9^ cells/L, a neutrophil count of 19.21 × 10^9^ cells/L, a lymphocyte count of 3.27 × 10^9^ cells/L, a platelet number of 642.60 × 10^9^/L, and a decreased hemoglobin level of 8.72 g/dL. The erythrocyte sedimentation rate was 65 mm/h, and C-reactive protein levels were 136.67 mg/L. The patient’s serum albumin level was 32.8 g/L. The level and rate of clearance of creatinine were 109 μmol/L and 45 mL/min, respectively. The serum IgG level was slightly elevated at 21.420 g/L, whereas IgA and IgM levels were normal. HIV autoantibodies were not detected. Serum and urine immunofixation electrophoresis were negative for monoclonal immunoglobulins. Tumor biomarkers were within their normal range. Computed tomography (CT) of the chest revealed a patchy dense shadow, suggesting left pleural effusion. Positron emission tomography/CT showed increased metabolic activity in the right lower lung, ninth rib on the left, and right ilium and multiple lymph nodes (right subclavicular region, mediastinum, and hilum) (Fig. 1c–e, j). Pathological examination of the lung, lymph nodes, subcutaneous nodule, and bone marrow (right ilium) showed no malignancy. Analysis of the lymph nodes and nodule revealed chronic purulent inflammation. Thus, he was diagnosed with community-acquired pneumonia (not excluding malignancy). He was administered anti-bacterial (ceftazidime for 5 days), anti-*Mycobacterium tuberculosis* (combination of isoniazid, rifampicin, pyrazinamide, and ethambutol for 2 weeks), and anti-non-*Mycobacterium tuberculosis* (clarithromycin + rifampin + ethambutol, for 10 days) therapy. However, his condition worsened, and he presented with multiple subcutaneous nodules with yellow secretion from the ulceration in the inguinal region and left thigh (Table 1).

**Table 1:** Laboratory results

Leukocytes (× 10^9^ cells/L)	25.43
Neutrophils (× 10^9^ cells/L)	19.21
Lymphocytes (× 10^9^ cells/L)	3.27
Hemoglobin (g/dL)	8.72
Platelets (× 10^9^/L)	642.60
erythrocyte sedimentation rate (mm/h)	65
C-reactive protein levels (mg/L)	136.67
Albumin (g/L)	32.8
Globulin (g/L)	35.9
aspartate aminotransferase (U/L)	58
alanine aminotransferase (U/L)	43
Creatinine (mol/L)	109
Creatinine clearance (mL/min)	45
immunoglobulin G (g/L)	21.420
immunoglobulin A (g/L)	3.809
immunoglobulin M (g/L)	1.005
percentage of CD4+ (%)	39.2
percentage of CD8+ (%)	31.3

*Reference ranges: leukocytes, 3.5–9.5 × 10^9^ cells/L; neutrophils, 1.8–6.3 × 10^9^ cells/L; lymphocytes, 1.1–3.2 × 10^9^ cells/L; platelets, 125–350 × 10^9^/L; hemoglobin, 13–17.5 g/dL; erythrocyte sedimentation rate, 0–15 mm/h; C-reactive protein levels, < 10 mg/L; creatinine, 59–104 µmol/L; Creatinine clearance, 85–125 mL/min; albumin, 40–55 g/L; globulin, 20–40 g/L; aspartate aminotransferase, 15–45 U/L; alanine aminotransferase, 9–60 U/L; immunoglobulin G, 8–18 g/L; immunoglobulin A, 0.9–4.5 g/L; immunoglobulin M, 0.84–1.32 g/L; percentage of CD4+, 30–46%; percentage of CD8+, 19.2–33.6%

**Fig. 2:**
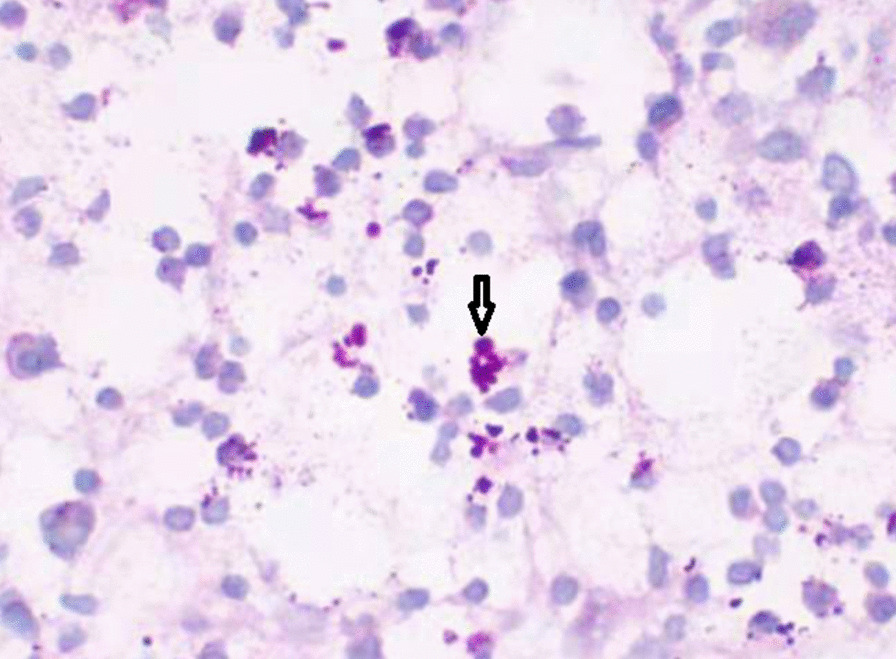
Pathological examination of subcutaneous nodules indicate that microorganisms were located outside the cells of the tissue and had a round, oval, or sausage-like shape. After periodic acid-Schiff staining, the microorganisms arranged in a mulberry-like shape, and a characteristic diaphragm with a sausage-like fungus could be observed (periodic acid-Schiff, magnification: ×400)

**Fig. 3:**
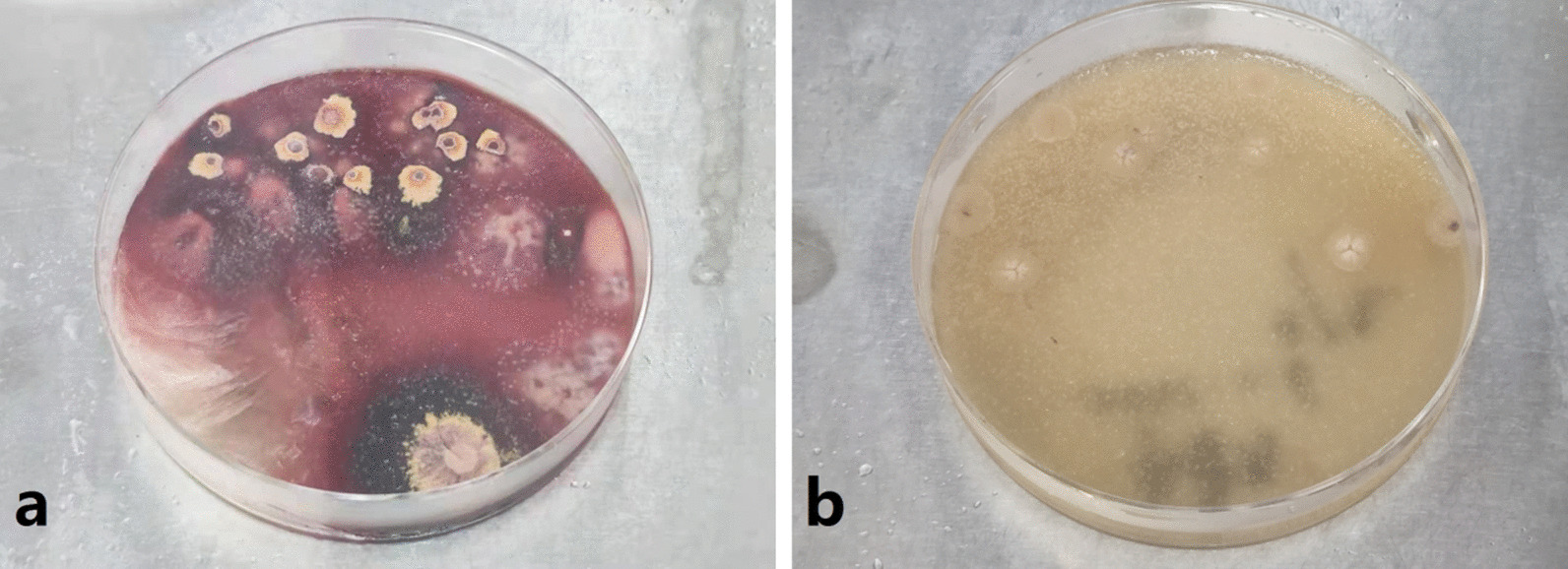
White to tan soft and smooth colonies were cultivated at 25 °C on Sabouraud dextrose agar (SDA), and red pigments permeated into the medium (**a**). A brown creamy yeast-like colony grew on SDA at 37 °C (**b**)

**Fig. 4:**
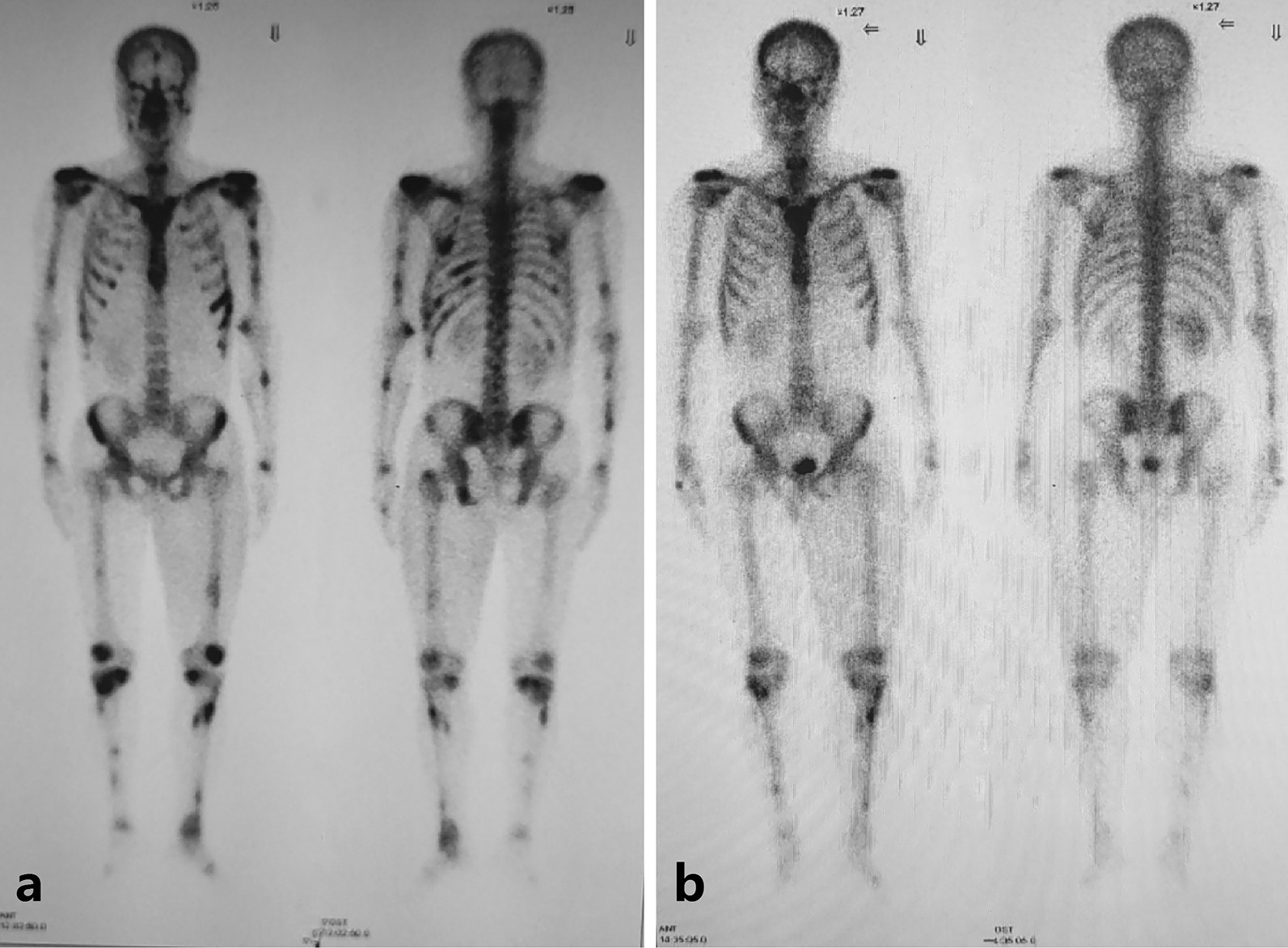
Findings after chemotherapy. Emission computed tomography (ECT) showed increased radioactive concentration by the clavicle, sternum, multiple ribs, vertebrae, right shoulder blade lower horn, pelvis, and long bones of the limbs. After chemotherapy (**a**), ECT showed reduced radioactive content in the primary bones (**b**)

**Fig. 5:**
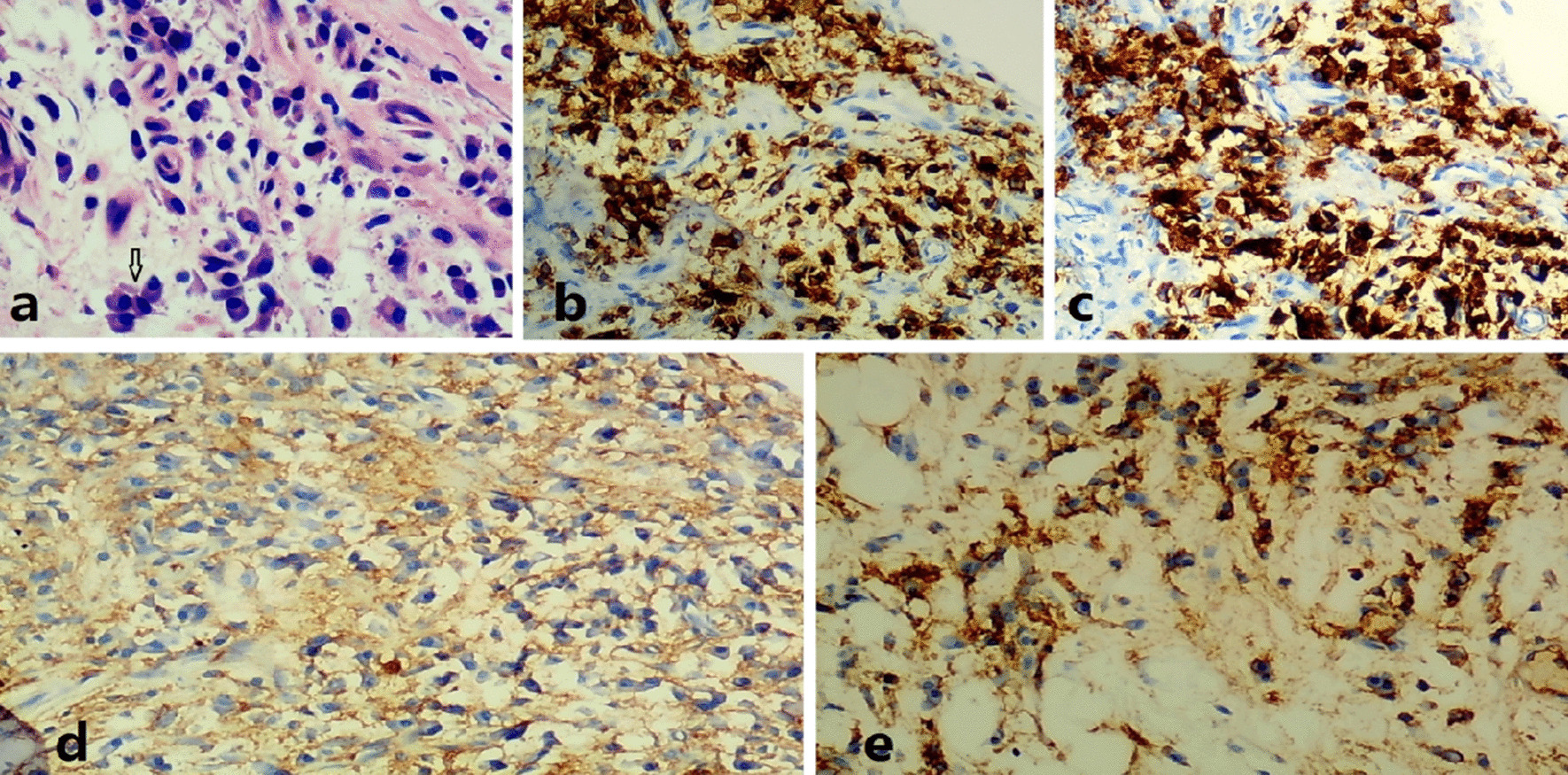
Bone marrow biopsy of the patient (hematoxylin & eosin staining, magnification: ×400). Neoplastic cells with prominent and eccentric nuclei occupied the bone marrow cavity, which had an abnormal morphology and were small, round, and spindle-shaped (**a**). Immunohistochemistry showing the presence of CD38 (**b**), CD138 (**c**), and kappa (**d**) and lambda (**e**) light chains (streptavidin-peroxidase staining, magnification: ×20)

Pathological examination of subcutaneous nodules indicated that microorganisms were located outside the cells of the tissue with a round, oval or sausage-like shape, after periodic acid-Schiff staining, the microorganisms arranged in a mulberry-like shape, and a characteristic diaphragm with a sausage-like fungus could be seen under the microscope at a 400-fold magnification by periodic acid-Schiff stain (Fig. 2). Then, we cultured the secretion from the subcutaneous nodules and observed the growth of a dimorphic pathogen on Sabouraud dextrose agar at 25 °C and 37 °C. At 25 °C, the microorganisms was a mold, growing as yellow-green colony and producing soluble red pigments that diffused into the agar, making the reverse side appear either pink or red (Fig. 3a). The microorganisms grew as a white yeast-like colony with radial stripes in the middle at 37 °C (Fig. 3b).The patient was re-diagnosed with disseminated TSM involving the lungs, lymph nodes, skin, bone, and subcutaneous tissue. He accepted fluconazole (400 mg/day, for 5 days) treatment at the beginning, but his symptoms were not relieved. His overall condition, except for the bone pain, improved after the antifungal amphotericin B treatment (starting at 10 mg/day, gradually increasing to 25 mg/day, for 2 weeks until the patient was discharged). Chest CT showed bone damage and cavities in the bone marrow of multiple ribs, clavicle, acromion and clavicle (Fig. 1f, g, i). Emission CT (ECT) showed a significant increase in the radioactive concentration of technetium-99 m-labeled bisphosphonates in the bilateral clavicles, sternum, multiple ribs, multiple vertebrae, inferior angle of the right scapula, pelvis, and long bones of the limbs, resulting in a dense morphology (Fig. 4a). Owing to the presence of osteolysis, we re-examined the bone marrow of the right ilium. Bone marrow smears suggested reactive hyperplasia and reduction in erythrocyte ratio. Bone marrow biopsy showed the presence of neoplastic cells with eccentric and prominent nucleoli in the bone marrow cavity (Fig. 5a). The cells exhibited abnormal morphology, having a small and round or spindle shape (Fig. 5a). Immunohistochemistry showed the expression of CD38 (Fig. 5b), CD138 (Fig. 5c), and kappa (Fig. 5d) and lambda (Fig. 5e) light chains which were consistent with the diagnosis of plasma cell myeloma. We conclusively diagnosed the patient with NSMM and disseminated TSM (in the lungs, lymph nodes, skin, and subcutaneous tissue). The patient was negative for TM infection and did not complain of bone pain after a year of chemotherapy (bortezomib, lenalidomide, and dexamethasone). ECT re-examination in 2017 showed a reduction in the radioactive content in primary bones (Fig. 4b).

## Discussion and conclusions

Recent studies have shown that an increasing population of TM-infected HIV-negative individuals have co-morbidities and are immunocompromised [1]. Hematological malignancies are cause by malignant cells and/or immunosuppressive therapy that enables severe immunodeficiency, easily causing opportunistic infections. For NSMM, plasma cells secrete soluble immune active molecules, spontaneously synthesize immunoglobulins without immune activity, and induce abnormal function in B, T, dendritic, and natural killer cells, thereby hindering the immune response in the body [4]. Traditional or novel chemotherapeutics damage the bone marrow, resulting in granulocytopenia and impaired immune function. So, myeloma patients are usually prone to opportunistic infections, mostly bacterial and viral ones, *Aspergillus*, *Candida albicans*, *Candida parapsilosis*, and *Scedosporium prolificans* infections have been reported [5], but TM infection is extraordinarily rare.

Symptoms associated with NSMM and TSM include fever, osteolysis, and swollen lymph nodes. Such patients present with osteolysis throughout the body that can be detected as well-defined lesions in imaging [6, 7]. Swollen lymph nodes are commonly found in patients with TSM and NSMM (upon extramedullary infiltration). Thus, it is difficult to differentially diagnose patients with NSMM and TM infection owing to the similarities in clinical manifestation and imaging, especially when the patient has swollen lymph nodes and/or osteolysis. We concluded that lung cancer in our patient could not be excluded, which was based on the multiple osteolysis observed during imaging. After TM infection was confirmed, we missed NSMM since the repeated pathological examination of the bone marrow showed no malignancy. Moreover, MM and TSM have similar serum globulin profiles; MM enhances the production of immunoglobulins, similar to TSM, especially that of IgG [8]. However, patients with NSMM do not exhibit heightened levels of the M-protein. For TSM patients presenting with multiple osteolysis and/or swollen lymph nodes, hematologic malignancies should be considered if active antifungal therapy proves to be ineffective. For patients with cancer, such as hematological malignancies and lung cancer [9], the presence of TM should be ascertained before or after chemotherapy. The final diagnosis primarily relies on multiple pathological examinations and the pathogenic culture of lymph nodes and bone marrow tissue.

The biopsy of lymph nodes in HIV-negative patients with TSM primarily exhibit granulomatous and purulent inflammation [10]. Granulomatous inflammation involves the presence of numerous epithelioid cells and multinucleated macrophages without caseous necrosis. Purulent inflammation is characterized by numerous neutrophils and macrophages involved in the phagocytosis of the fungus with varying degree of tissue necrosis and abscess. Hematoxylin and eosin staining showed pale blue granules in the fungal cytoplasm. Periodic acid-Schiff or Wright–Giemsa staining revealed of clustered or scattered fungi in the lymphoid tissues; the fungi are distributed in or free from macrophages [11]. Culture of lymph node tissues was performed on Sabouraud dextrose agar, TM was grown at 25 °C and 37 °C. At 25 °C, TM produced a soluble red pigment that diffuses into the agar. At 37 °C, it resembled yeast-like growth with a spherical, oval, or sausage-like form and a characteristic transverse septum in the middle [12]. In general, pathological examination of lymph nodes in patients with NSMM shows the destruction of the node architecture without inflammatory cell infiltration, which is replaced by a major population of myeloma plasma cells. Bone marrow smears from patients with TSM show the presence of actively proliferating cells. TM-specific staining shows the presence of the fungus in or outside macrophages. Bone marrow cultures may be positive for TM. Bone marrow smears and biopsies from patients with NSMM show a large number of myeloma plasma cells with enlarged eccentric nucleoli. Immature plasma cells have mono-, poly-, or segmented nuclei with prominent nucleoli [13].

Since TM infection is rarely found in patients with NSMM, there is no standardized treatment. Thus, it is imperative to devise novel efficacious therapeutic strategies. Therapy of HIV-negative TSM patients with osteolysis is long, patients frequently relapse [7], or new osteolysis appears. The current treatment of HIV-positive TSM patients involves amphotericin B (0.7–1 mg/kg/day for 2 weeks) followed by itraconazole (200 mg twice daily for 10 weeks) [14]. For newly diagnosed myeloma patients, the National Comprehensive Cancer Network recommends that, regardless of transplantation, the VRd (bortezomib/lenalidomide/dexamethasone combination therapy) chemotherapeutic regimen can improve tolerability and efficacy [15]. Myeloma treatment requires long-term chemotherapy. However, most chemotherapeutics result in immunosuppression and enhance the risk of opportunistic infections. Therefore, the duration of chemotherapy should be tailored for patients based on the presence of TSM. Chemotherapy suppresses immune function, thereby aggravating the infection. Thus, for TM infected patients with malignant tumors that are not in remission, choosing the time of chemotherapy is important. We have noticed that antifungal therapy followed by the VRd regimen in newly diagnosed myeloma patients with TM infection proves to be more effective in controlling symptoms. During treatment, symptoms should be carefully monitored to determine the recurrence of TSM or progression of myeloma.

## Data Availability

All data generated or analyzed during this study are included in this published article.

## Abbreviations

NSMM: Non-secretory myeloma
TSM: Talaromycosis
TM: *Talaromyces marneffei*
HIV: Human immunodeficiency virus
MM: Multiple myeloma
CT: Computed tomography
ECT: Emission CT
Ig: Immunoglobulin
